# Enrichment of milk with magnesium provides healthier and safer dairy products

**DOI:** 10.1038/s41522-017-0032-3

**Published:** 2017-10-11

**Authors:** Noa Ben-Ishay, Hilla Oknin, Doron Steinberg, Zipi Berkovich, Ram Reifen, Moshe Shemesh

**Affiliations:** 1 Department of Food Quality and Safety, Institute of Postharvest Technology and Food Sciences, Agricultural Research Organization (ARO) the Volcani Center, 7528809 Rishon LeZion, Israel; 2 The Robert H. Smith Faculty of Agriculture, Food and Environment, Institute of Biochemistry, Food Science and Nutrition, The Hebrew University of Jerusalem, 76100 Rehovot, Israel; 3 Biofilm Research Laboratory, Institute of Dental Sciences, Faculty of Dental Medicine, Hebrew University-Hadassah, Jerusalem, Israel

## Abstract

Biofilms on the surfaces of milk-processing equipment are often a major source of contamination of dairy products. Members of the genus *Bacillus* appear to be among the most commonly found bacteria in dairy farms and processing plants. *Bacillus* species may thrive in dairy farm equipment and in dairy products since they can form robust biofilms during growth within milk. We found that fortification of milk with magnesium mitigated biofilm formation by *Bacillus* species, and thus could notably reduce dairy product spoilage. We also show that the mode of action of Mg^2+^ ions is specific to inhibition of transcription of genes involved in biofilm formation. Our further findings indicate that in the presence of Mg^2+^ bacterial cells are hypersensitive to the heat pasteurization applied during milk processing. Additionally, we demonstrated that enrichment of milk with magnesium improved technological properties of milk products such as soft cheeses. Finally, we report that there is a notable increase in the intestinal bioavailability potential of magnesium from supplemented milk compared with that from non-supplemented milk.

## Introduction

Bacterial contamination can adversely affect the quality, functionality, and safety of milk and its derivatives. It appears that the major source of the contamination of dairy products is often associated with biofilms on the surfaces of milk processing equipment.^1^ Members of the genus *Bacillus* are among the most commonly found bacteria in dairy farms and processing plants.^2,3^ *Bacillus* species may thrive in dairy farm equipment and in dairy products because they can form robust biofilms during growth within milk.^4^ Biofilm is a highly structured multicellular community, which enables bacteria to survive in hostile environments.^5^ Biofilm formation is a multistage process in which cells produce an extracellular matrix that is typically composed of polysaccharides, proteins, and nucleic acids.^6^ These exopolymeric substances may surround and protect the bacteria.^7^ Thus, biofilm bacteria are considered to be more resistant than planktonic cells to various antimicrobials.^8^ Biofilms being the potential sources of contamination can also increase corrosion rates of metal pipes and equipment often used in the milk industry. In addition, existence of biofilm within the equipment may impair heat transfer and can increase fluid frictional resistance.^9^

Dairy products form one of the leading sectors impacted by food loss, as nearly 20% of conventionally pasteurized fluid milk is discarded and thereby lost to consumption each year.^10,11^ Bovine milk is highly nutritious, and this makes it an ideal medium for growth of microorganisms; it contains abundant quantities of water and nutrients (such as lactose, proteins, and lipids) and has a nearly neutral pH. Since microorganisms in milk may present spoilage and/or health risks, milk manufacturing is subject to extremely stringent regulations^12^ but nevertheless, some bacteria, especially the *Bacillus* species, are able to overcome these obstacles. For instance, thermophilic and spore-forming bacteria are able to survive pasteurization procedures, and psychrotrophic bacteria thrive at the low temperatures at which milk is stored.^3^ Moreover, bacterial spores can survive treatment with reagents commonly used in a cleaning-in-place procedures, which include regular cleaning of processing equipment, usually with alkaline and acidic liquids at high temperatures.^13,14^ In addition, according to our recent study, *Bacillus* species form biofilm-related structures termed bundles during their growth in milk.^4^ This biofilm-associated phenomenon, which appears to be highly conserved in *Bacillus* species,^4^ may have tremendous undesirable implications in the dairy industry. Some of these bacteria produce enzymes—proteases and lipases—that cause off-flavors and curdling in the final product.^10,15^

Therefore, a range of antimicrobial strategies to control biofilms have been proposed. Several studies have reported on development of sensors for identifying biofilms in their initial stages,^16^ in order to prevent the maturation of biofilms. Other studies have tried to identify materials that would prevent formation of biofilms.^17^ However, conventional cleaning and disinfection regimes and antimicrobial strategies may be ineffective in controlling biofilm formation and dissemination of resistance.^3^ We recently reported that magnesium ions (Mg^2+^) affected biofilm formation via down-regulation of the expression of extracellular matrix genes,^18^ which suggests that the molecular mechanism behind the inhibitory effect of Mg^2+^ ions is primarily related to expression of matrix genes. In the present study, we investigated the potent inhibitory activity of Mg^2+^ ions on biofilm formation by *Bacillus* species within milk, and found that bacteria became more sensitive to processing procedures of dairy food, e.g., heat-treatment pasteurization, in the presence of Mg^2+^ ions. We also showed that Mg^2+^ ions could improve technological properties of milk products, e.g., by promoting clotting in milk and enhancing protein contents in cheeses.

## Results and discussion

**Fig. 1:**
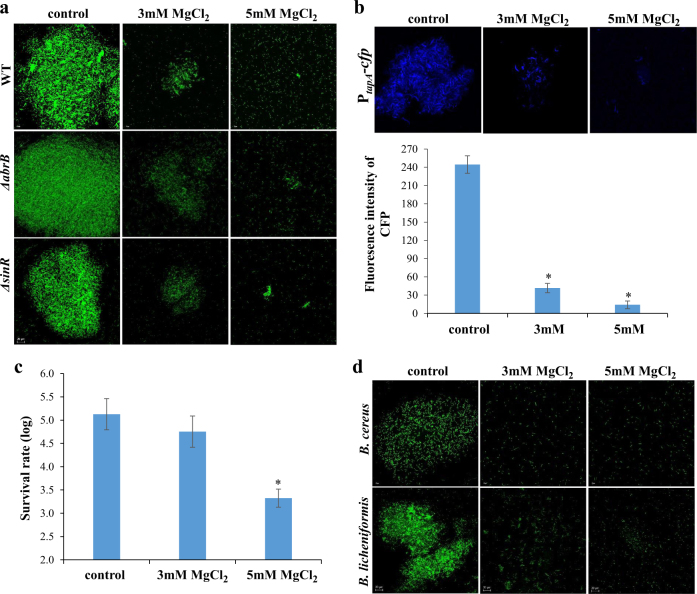
Antimicrobial effect of Mg^2+^ ions on *Bacillus* species during growth within milk. **a** The effect of Mg^2+^ ions on biofilm bundles formation by fluorescently tagged *B. subtilis* strains of the wild type (WT), the Δ*abrB* mutant, and the Δ*sinR* mutant. Bacterial cells were grown in milk with or without addition of MgCl_2_, and then were analyzed using confocal microscope. **b** The effect of Mg^2+^ ions on transcription of *tapA* operon that is responsible for the matrix production in *B. subtilis*. The WT cells harboring P_*tapA*_-*cfp* transcriptional fusion were grown overnight in milk with or without addition of MgCl_2_ and analyzed using confocal microscope. **c** The survival rate of *B. subtilis* under heat treatment within milk in the presence of Mg^2+^ ions. Bacterial cells grown in milk (with or without addition of MgCl_2_), were subjected to heat treatment (performed at 63 °C for 3 min) and the survival rate was determined using the CFU method. **d** The effect of Mg^2+^ ions on biofilm formation by different *Bacillus* species known to contaminate milk. The cells of *B. cereus* ATCC 10987 and *B. licheniformis* MS310 in milk (with or without addition of MgCl_2_) were stained with the LIVE/DEAD viability kit and analyzed using confocal microscope. *Scale bar*—20 µm. *P-value < 0.05 for comparison with control unsupplemented milk. Error bars represent standard deviation (SD)

Our initial results indicated that at elevated concentrations Mg^2+^ ions could inhibit biofilm formation by *Bacillus subtilis* during growth within milk. We visualized the effect of Mg^2+^ ions microscopically by testing a bundling phenotype of fluorescently tagged *B. subtilis* cells (YC161 with P_*spank*_-*gfp*), which produce GFP constitutively. As seen in Fig. 1a, Mg^2+^ ions notably inhibited formation of biofilm bundles in a concentration-dependent manner. Remarkably, though enrichment of milk with MgCl_2_ up to 3 mM slightly affected biofilm formation, at a concentration of 5 mM and higher biofilm formation was completely inhibited (Fig. 1a and Supplementary Fig. S1). This result confirms the potential of Mg^2+^ ions to inhibit biofilm formation by *B. subtilis* during its growth within milk. To confirm that the inhibition of biofilm formation by Mg^2+^ ions was not a result of toxicity to bacterial cells, we tested the effects of various concentrations of Mg^2+^ ions on bacterial growth. As shown in Supplementary Fig. S2, growth curve analysis suggested that there was no significant effect of Mg^2+^ ions on bacterial growth at the tested concentrations. Hence, we conclude that the mode of action of Mg^2+^ ions specifically relates to inhibition of biofilm formation.

In *B. subtilis*, biofilm formation depends on synthesis of extracellular matrix, whose production is specified by two major operons: the *epsA-O* and *tapA-sipW-tasA* operons.^19–21^ The *epsA-O* operon is responsible for production of the exopolysaccharides, and the *tapA* operon for production and assembly of an amyloid-like fibers.^22,23^ We hypothesized that the dramatic decrease in biofilm formation in the presence of Mg^2+^ ions could be due to down-regulation of those genes involved in matrix synthesis. To test this hypothesis, we analyzed the effect of Mg^2+^ on matrix gene expression by using transcriptional fusion of the promoter for *tapA* to the gene coding for cyan-fluorescent protein (P_*tapA*_-*cfp*). As shown in Fig. 1b, expression of the *tapA* operon was notably reduced in response to the addition of Mg^2+^ ions. In addition, we performed real-time reverse transcription polymerase chain reaction (RT-PCR) analysis to quantify the effect of Mg^2+^ ions on the expression of the *epsH* and *tasA* genes of the matrix operons. The results show that there is a significant reduction in the expression of these genes as a response to addition of Mg^2+^ ions (Supplementary Fig. S3). In overall, these results suggest that addition of Mg^2+^ ions down-regulates synthesis of the extracellular matrix by *B. subtilis*.

Since biofilm formation is often considered as a protective mode of growth, we hypothesized that during growth within milk in the presence of Mg^2+^ ions, bacteria would be less protected against processing procedures such as heat-treatment pasteurization. Therefore, we tested the survival, following heat treatment, of *B. subtilis* cells within milk supplemented with Mg^2+^ ions. As shown in Fig. 1c, the bacteria were hypersensitive to heat treatment in the presence of Mg^2+^ ions. We found that there was about two-log reduction in survival rate of bacterial cells in the presence of Mg^2+^ ions compared with that in the control unsupplemented milk (Fig. 1c). Interestingly, our additional data suggest that Mg^2+^ ions could not notably affect the sporulation rate by *B. subtilis* (data not shown). Therefore, the most likely explanation for this finding is that in the presence of Mg^2+^ ions there was down-regulation of production of the extracellular matrix, which may serve as a protective shield for bacterial cells. To further confirm the assumption that reduced survival of the cells depends on biofilm formation, we tested the survival of the *∆eps∆tasA* double mutant (which is unable to form biofilm) during heat treatment. We found about 5-fold reduction in the survival ability of the mutant compared to the wild type (Supplementary Fig. S4). Therefore, we assume that the survival ability during heat treatment depends on the extracellular polymeric substances, which can serve as a protective substance for biofilm bacteria.

In *B. subtilis*, the extracellular matrix operons, *tapA* and *epsA-O*, are under the direct negative control of two repressors, SinR and AbrB, which act synergistically to repress the matrix-encoding genes.^21,22^ Derepression is triggered by the transcriptional regulator, Spo0A which is activated by phosphorylation.^24^ Spo0A phosphorylation is controlled by members of the Kin family, which include five histidine kinases (KinA–E),^25,26^ which respond to various environmental and physiological cues.^27,28^ In light of our finding that Mg^2+^ ions downregulated expression of the extracellular matrix genes in *B. subtilis*, we considered that the molecular mechanism for the inhibitory effect of Mg^2+^ ions could be primarily related to the Kin-Spo0A pathway. It is conceivable that Mg^2+^ ions could affect directly transcription of the extracellular matrix operons controlled by this pathway. We therefore tested the effect of Mg^2+^ ions on biofilm formation by the ∆*abrB* or ∆*sinR* mutant strains, which do not express the AbrB and SinR repressor proteins, respectively. Given that Mg^2+^ ions inhibit formation of the biofilm bundles in these mutants, it could be suggested that Mg^2+^ ions affect the signaling pathway by interfering the transcription of the matrix operons. As seen in Fig. 1a, Mg^2+^ ions indeed prevented formation of the biofilm bundles in these mutant strains. Thus, one possible explanation of this result is that Mg^2+^ ions act downstream to AbrB and SinR in the Kin-Spo0A signal transduction pathway; therefore, they probably prevent formation of biofilm bundles by interfering directly with transcription of the matrix operons.

We also investigated whether Mg^2+^ ions-mitigated formation of biofilm bundles in other *Bacillus* species that are known to contaminate milk. For this, we tested strains of the pathogenic *B. cereus*, and also of the nonpathogenic *B. licheniformis*, both of which have been shown to contaminate milk.^13,29^ As shown in Fig. 1d, the inhibitory effect of Mg^2+^ ions is conserved in other *Bacillus* species too, though growth curve analysis suggested that there was no significant effect of Mg^2+^ ions on bacterial growth at the tested concentrations (Supplementary Fig. S2).

**Fig. 2:**
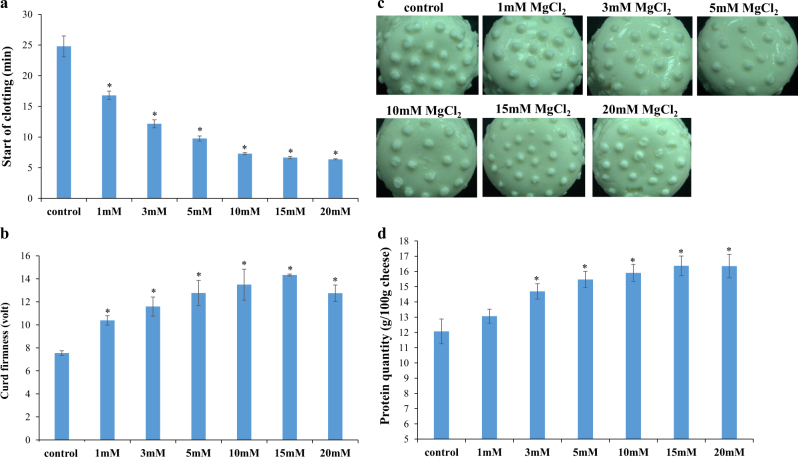
Effects of Mg^2+^ ions on technological properties of dairy products. Effects of Mg^2+^ ions on milk-clotting parameters were tested with the Optigraph instrument, which enables **a** detection of the start of the clotting process, and **b** determination of the firmness of the obtained curd. **c** Visualization of soft cheese samples prepared from milk supplemented with MgCl_2_ compared with those from unsupplemented milk. **d** Effect of Mg^2+^ ions on incorporation of protein into soft cheese. The cheeses preparation and the detection of the level of protein have been performed as described in Materials and methods. *P-value < 0.05 for comparison with control unsupplemented milk. Error bars represent standard deviation (SD)

In light of the common knowledge and long-standing reports that minerals can form complexes with milk components, such as proteins and peptides,^30^ we tested the effects of Mg^2+^ ions on some technological properties of milk products. In essence, we examined the effects of various concentrations of Mg^2+^ on milk-clotting parameters such as start of clotting (minutes) and curd firmness (V) after 60 min, which were measured with the Optigraph instrument (Ysebaert, Frepillon, France). As shown in Fig. 2a, clotting started significantly earlier in milk supplemented with MgCl_2_ than in unsupplemented milk. Moreover, the curd firmness was notably higher in the supplemented samples (Fig. 2b). These results indicate that the curdling process appeared to be much improved in the presence of MgCl_2_. Furthermore, by supplementing milk with MgCl_2,_ we examined the effects of various concentrations of Mg^2+^ ions on soft cheese properties and on incorporation of milk proteins into the cheese (Fig. 2c, d). By using the Kjeldahl method for measuring the level of protein in cheese, we found an increase of about 4 g in incorporated protein (per 100 g cheese prepared from supplemented milk), compared with that in a cheese prepared from unsupplemented milk (Fig. 2d). In fact, there is an increase of about 33% in the protein concentration in the magnesium-enriched cheese, compared to unfortified cheese. This result indicates that a lesser amount of protein is lost during cheese preparation from magnesium-supplemented milk, leading to improving in cheese quality with significantly higher protein quantity.

**Fig. 3:**
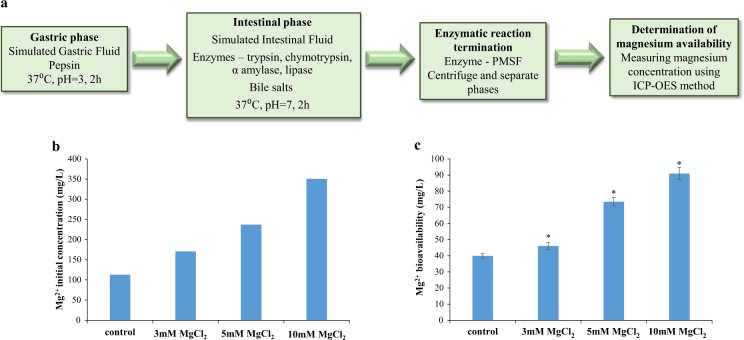
Enrichment of milk with magnesium leads to increase in the bioavailability potential of magnesium from milk. **a** Schematic representation of static in vitro digestibility model in which gastric and small intestine digestion are mimicked in two consecutive steps, enabling analysis of available Mg^2+^ ions following digestion. **b** Baseline—initial concentration of Mg^2+^ ions in milk samples before digestion. **c** Bioavailability potential—Mg^2+^ ions concentration after in vitro digestive process of the milk samples. The concentration of Mg^2+^ ions has been determined as described in Materials and methods. *P-value < 0.05 for comparison with control unsupplemented milk. Error bars represent standard deviation (SD)

Notably, recently magnesium has attracted increasing attention in the functional foods industry, and several clinical studies have highlighted the beneficial effects of this important mineral on human health;^31^ it appears that magnesium is involved in hundreds of enzymatic reactions in the human body. This cation plays important roles in the physiological functioning of the brain, heart, and skeletal muscles, and has anti-inflammatory properties.^31^ Significant amount of Mg^2+^ ions is stored in bone, where they bind at the surface of the hydroxyapatite crystals. Presence of Mg^2+^ ions increases the solubility of P_i_ and Ca^2+^ hydroxyapatite and thereby acts on the crystal size and formation.^31^ Moreover, Mg^2+^ ions stimulate osteoblast proliferation, suggesting that magnesium deficiency results in decreased bone formation.^31^ Magnesium deficiency impacts on the bone also indirectly by affecting the homeostasis of the two master regulators of calcium homeostasis, i.e., parathyroid hormone and 1,25(OH)_2_-vitamin D thus leading to hypocalcemia.^32^ Hence, magnesium together with calcium are important supplemented elements for building and strengthening the bones. Since calcium is regularly available in milk products, the addition of magnesium may complete the daily intake of these important minerals. Nonetheless, recent reports estimate that at least 60% of Americans do not consume the recommended daily amount of magnesium.^33^ Therefore, we wished to determine the bioavailability potential of magnesium from milk supplemented with MgCl_2_, by using a static in vitro digestibility model in which gastric and small intestine digestion is mimicked in two consecutive steps, enabling analysis of available Mg^2+^ ions following digestion.^34^ As shown in Fig. 3, there was a two-fold increase in the bioavailability potential of magnesium from milk supplemented with either 5 mM or 10 mM MgCl_2_, compared with its bioavailability potential from unsupplemented milk.

The key finding of our study is that Mg^2+^ ions mitigated formation of biofilm bundles within milk. This phenomenon was found to be conserved in *B. subtilis*, *B. cereus*, and also *B. licheniformis*, all of which are known to contaminate milk. Thus, we speculate that the ability of Mg^2+^ ions to inhibit biofilm formation could be a universal phenomenon that is common to a wide range of bacteria, particularly *Bacillus* species. We also found that Mg^2+^ ions may act downstream to AbrB and SinR in the Kin-Spo0A signaling pathway, therefore Mg^2+^ ions probably prevent formation of biofilm bundles by interfering with transcription of the extracellular-matrix encoding genes.

The existence of biofilms within milk as well as on milk-contacting surfaces appears to present tremendous implications in the dairy industry: biofilm-forming bacteria might adhere to surfaces of milking equipment, or perhaps circulate through the milking pipelines and get trapped in the filters within the milking system. A key feature of a biofilm is that it provides protection for the vegetative bacteria as well as for spores within it.^4^ Therefore, the findings of this study regarding mitigation of biofilm formation opens opportunities for important innovations in the food industry; for instance, development of useful strategies to control microbial food spoilage in order to improve food safety and quality. In this context, we further speculate that hypersensitivity of bacteria to heat treatment in the presence of Mg^2+^ ions will lead to possible improvement in microbial quality of other food products, for instance during liquid food production such as fruit and vegetable juices.

In conclusion, enrichment of dairy foods with magnesium provides a straightforward approach to improving the microbial quality as well as the technological properties of dairy products, and offers an important means to deliver this important mineral to humans.

## Methods

### Strains and growth media

The *B. subtilis* strains used in this study are listed in Supplementary Table S1, in the Supplementary Material file. For routine growth, all strains were propagated in Lysogeny broth (LB) comprising (per liter): 10 g of tryptone, 5 g of yeast extract, and 5 g of NaCl, or on solid LB medium supplemented with 2% agar. For starter cultures, bacteria were grown to early stationary phase in LB medium for 5 h at 37 °C with shaking at 150 rpm. For generation of biofilm bundles, the starter cultures were dilited 1:100 into UHT milk (Tnuva, Rehovot, Israel) and incubated overnight at 30 °C with shaking at 25 rpm. To test the effect of magnesium ions on biofilm formation, the freshly prepared 1 M solution of MgCl_2_ (Merck KGaA, Darmstadt, Germany) was added directly to the milk at various concentrations.

### Confocal laser scan microscopy (CLSM) analysis

For visualizing *B. subtilis* biofilm bundles, we used the strain YC161, which produced GFP constitutively (P_*spank*_-*gfp*).^35^ The strain was first grown to early stationary phase for 5 h at 37 °C in shaking culture at 150 rpm, in LB medium. Next, 200 µL of suspension from the resulting culture were introduced into 20 mL of UHT milk, supplemented or not with MgCl_2_, and incubated overnight at 30 °C with shaking at 25 rpm. Afterwards, 1 mL of suspension from each sample was collected and centrifuged at 10,000 rpm for 2 min. The supernatant was removed and the cells were washed with sterile distilled water and then suspended in 50 μL of distilled water. For microscope observation, 8 μL of each sample were transferred onto glass slides and visualized with a SP8 CLSM (Leica, Wetzler, Germany) equipped with a HC PL APO 40x/1.1 water immersion objective (Leica, Wetzlar, Germany) and 488 nm laser for GFP excitation or 458 nm laser for CFP excitation. For visualizing the expression of the *tapA* operon, we used *B. subtilis* strain YC189, which harbors a gene coding to CFP under the control of the *tapA* promoter (P_*tapA*_-*cfp*).^22^ Fluorescence intensity of cells expressing the *tapA* operon was estimated by calculating the average pixel intensity from each successive focal plane by means of Mica software (Multi-Image Analysis, CytoView, Petah-Tikva, Israel). For experiments with *B. cereus* or *B. licheniformis*, the cells were stained with the FilmTracer LIVE/DEAD Biofilm Viability Kit (Molecular Probes, Eugene, Oregon, USA) according to the manufacturer’s instructions. Fluorescence emission from the stained samples was measured with a SP8 CLSM (Leica) equipped with 488 and 552 nm lasers.

### RNA extraction and reverse transcription real-time PCR

To quantify the effect of magnesium ions on expression of genes of the matrix operons (*epsA-O* and *tapA-sipW-tasA*), we used the real-time RT-PCR method. Initially, *B. subtilis* cells were grown to early stationary phase for 5 h at 37 °C in shaking culture at 150 rpm, in LB medium. For generation of biofilm bundles, 200 µL of suspension from the resulting culture were introduced into 20 mL of LB supplemented with 3% lactose similarly as described recently.^36^ To investigate the effect of magnesium ions, MgCl_2_ was added to the growth medium to final concentration of 5 mM. The samples were incubated overnight at 30 °C with shaking at 25 rpm. Next, one milliliter from each samples was collected and centrifuged at 5000 rpm for 5 min. The RNA was harvested using the GenUP Total RNA Kit (Biotechrabbit, Hennigsdorf, Germany), according to the manufacturer protocol. RNA concentration was determined spectrophotometrically using the Nanodrop-2000 Instrument (ThermoFisher Scientific, Waltham, Massachusetts). 1 µg of RNA was used as a template in reverse transcription reactions carried out using qScript cDNA Synthesis Kit (Quantabio, Beverly, Massachusetts), according to the manufacturer protocol. The cDNA samples were stored at −20 °C until used. The corresponding oligonucleotides primers were designed using the Primer3 plus software (http://www.bioinformatics.nl/cgi-bin/primer3plus/primer3plus.cgi) and manufactured by IDT. For each primer set, a standard amplification curve was plotted (critical threshold cycle against log of concentration) and only those with slope ≈ –3.5 were considered reliable primers. The critical threshold cycle (*C*_t_) was defined as the cycle in which fluorescence becomes detectable above the background fluorescence and is inversely proportional to the logarithm of the initial number of template molecules. The real-time PCR reaction mixture (15 μL) contained 7.5 μL PerfectCTa SYBER Green FastMix (Quantabio, Beverly, Massachusetts), 1.75 μL sterile distillated water, 5 μL of the cDNA sample, and 0.25 μM of the appropriate forward and reverse polymerase chain reaction (PCR) primers. PCR conditions included an initial denaturation at 95 °C for 15 min, followed by a 40-cycle amplification consisting of denaturation at 95 °C for 10 s, annealing at 5 °C for 20 s and extension at 72 °C for 20 s. RNA samples, without addition of reverse transcriptase, were used as a negative control in order to determine whether the RNA samples were contaminated by residual genomic DNA. The expression levels of the tested genes were normalized using *sigA* gene of *B. subtilis* as an internal standard. The primers used for real-time RT-PCR are listed in Supplementary Table S2.

### Growth curve analysis

The following strains were used for growth analysis: NCIB3610 of *B. subtilis*,^37^ *B. cereus* ATCC 10987 and *B. licheniformis* MS310. Initially, the cells were grown overnight in LB at 23 °C with shaking at 90 rpm. In the morning the cultures were diluted 1:100 into ultra-high temperature (UHT) processed milk (Tnuva), with or without addition of various concentrations of MgCl_2_, and incubated for 6 h at 37 °C with shaking at 150 rpm. Every 1.5 h, 1 mL of each sample was collected and the number of viable cells was determined by the CFU method, i.e., serial dilutions from each sample were prepared, spread-plated on LB agar and incubated overnight at 37 °C, and colonies were counted.

### Survival of *B. subtilis* under heat treatment

To analyze the survival of bacteria following heat treatment within milk supplemented with MgCl_2_, we used the NCIB3610 strain of *B. subtilis*.^37^ The strain was first grown to stationary phase overnight in LB medium at 23 °C with shaking at 90 rpm. In the morning the cultures were diluted 1:100 in UHT milk (Tnuva), with or without addition of various concentrations of MgCl_2_, and incubated for 6 h at 37 °C with shaking at 25 rpm, for generation of biofilm bundles. The samples then were heat treated for 3 min at 63 °C in a water bath, and mildly sonicated for 20 s—amplitude, 20%; pulse, 10 s; pause, 10 s—with the Ultrasonic processor (Sonics, Newtown, USA). It is believed that different cell types (as vegetative cells as well as spores) can survive the heat treatment performed in this experiment. The number of surviving cells after heat treatment was quantified by the CFU method, as described in the previous paragraph. In order to confirm that the reduction in the survival of *B. subtilis* during heat treatment in the presence of magnesium ions is related to inhibition of biofilm formation, we decided to perform the heat treatment experiment (as described above) on *B. subtilis* double mutant strain (Δ*epsH*Δ*tasA*) too. This mutant is defective in structural genes encoding the extracellular matrix components, which is essential for biofilm formation.

### Milk clotting analysis

We tested milk-clotting parameters, such as time to start of clotting (min) and curd firmness (V) after 60 min, in the presence of various concentrations of MgCl_2_, by means of the Optigraph instrument (Ysebaert, Frepillon, France) as described previously.^38^ Milk was obtained from the dairy farm of the Agricultural Research Organization (ARO), Rishon LeZion, Israel.

### Protein quantity analysis in soft cheeses

Milk was obtained from the dairy farm of the ARO, and cheeses were prepared from 50 mL of milk by using Rennet enzyme (Gist-Brocades, Delft, Netherlands) with or without addition of various concentrations of MgCl_2_. Initially, the Rennet was diluted 1:100 in distilled water, and 2.5 mL of the diluted enzyme were added to each sample. The samples were incubated in a water bath at 30 °C for 1 h. The resulting cheeses were cut and warmed at 40 °C for 30 min in a water bath, in order to drain the whey; then they were put into perforated tubes and kept at 4 °C overnight to remove the whey. The level of protein has been determined by the Kjeldahl method as described previously.^39^

### Evaluation of magnesium bioavailability potential from milk by using a static in vitro digestion method

We determined the bioavailability potential of magnesium from milk supplemented with MgCl_2_, by using a static in vitro digestibility model in which gastric and small intestine digestion are mimicked in two consecutive steps, enabling analysis of available magnesium ions following digestion.^34^ Initial magnesium concentration in the milk samples, and magnesium concentration after digestion, i.e., bioavailability potential, were analyzed with the ARCOS inductively coupled plasma optical emission spectrometry system (Spectro GmbH, Kleve, Germany), as described previously.^40^

### Statistical analysis

The results were subjected to either Student’s *T*-test or one way analysis of variance at a significance level of *P < *0.05, to compare the control and tested samples. The results are based on three biological repeats performed in duplicates.

### Data availability statement

The data sets generated during and/or analyzed during the current study (which are not included in this published article or its supplementary information files) will be available from the corresponding author on reasonable request.

## Electronic supplementary material

Supplemental Material
