# Supplementary material for: Enrichment of milk with magnesium provides healthier and safer dairy products
Source: NPJ Biofilms Microbiomes. 2017 Oct 11;3:24. doi: 10.1038/s41522-017-0032-3 (PMC5636824; doi:10.1038/s41522-017-0032-3)
Supplement: Supplementary file 1 — Supplemental Material [file 41522_2017_32_MOESM1_ESM.pdf]

Supplementary material

Enrichment of milk with magnesium provides healthier and safer dairy products

Noa Ben-Ishay[1,2], Hilla Oknin[1,3], Doron Steinberg[3], Zipi Berkovich[2], Ram Reifen[2], Moshe Shemesh[1*]

[1]Department of Food Quality and Safety, Institute of Postharvest Technology and Food Sciences, Agricultural Research Organization (ARO) the Volcani Center, Rishon LeZion, Israel.

[2]The Robert H. Smith Faculty of Agriculture, Food and Environment, the Institute of Biochemistry, Food Science and Nutrition, the Hebrew University of Jerusalem, Rehovot, Israel.

[3]Biofilm Research Laboratory, Institute of Dental Sciences, Faculty of Dental Medicine, Hebrew University-Hadassah, Jerusalem, Israel.

[*]Correspondence should be addressed to: Moshe Shemesh, Department of Food Quality and Safety, Institute for Postharvest Technology and Food Sciences, ARO the Volcani Center, Derech Hamacabim, POB 15159, Rishon LeZion 7528809, Israel; Email: moshesh@agri.gov.il.

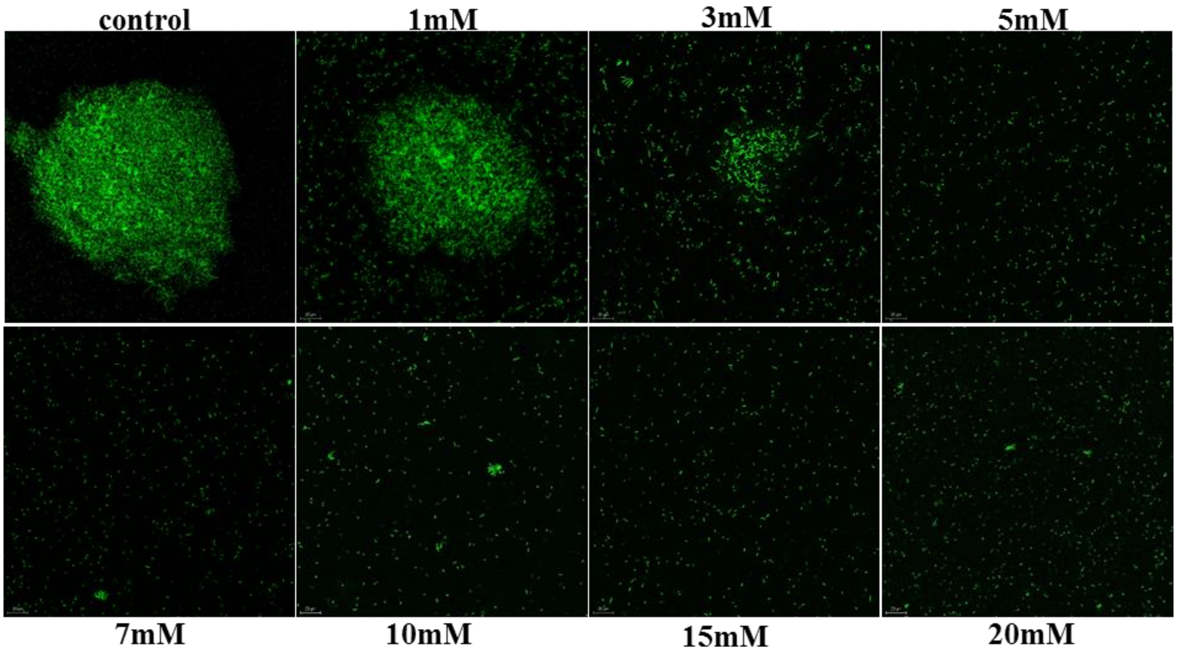

Figure S1 | Effect of Mg^{2+} ions on biofilm formation by *B. subtilis* during growth in milk. Fluorescently tagged *B. subtilis* cells grown in milk in the presence of $MgCl_2$ at various concentrations were analyzed using confocal microscope. Scale bar – 20 µm.

(a)

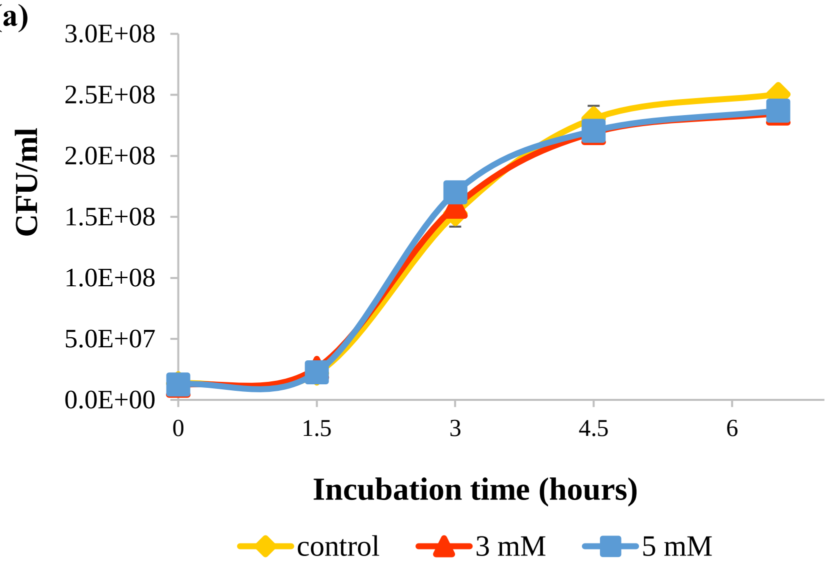

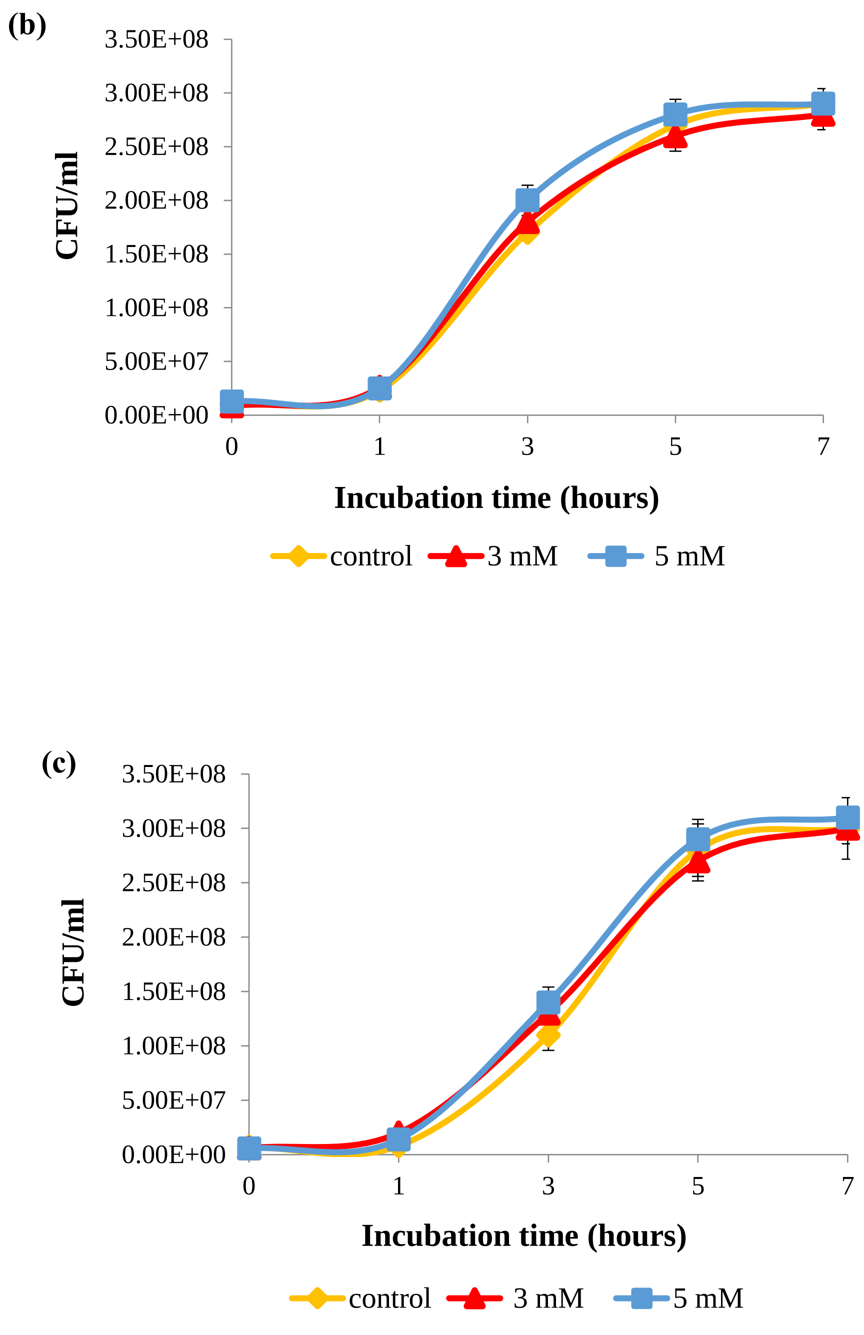

Figure S2 | Effect of Mg^{2+} ions on bacterial growth within milk. Growth curves were analyzed for (a) *B. subtilis*, (b) *B. cereus* and (c) *B. licheniformis* cells incubated in milk,

in the presence of various concentrations of $MgCl_2$ and subjected to viable cell counts using the CFU method. Error bars represent standard deviation (s.d.).

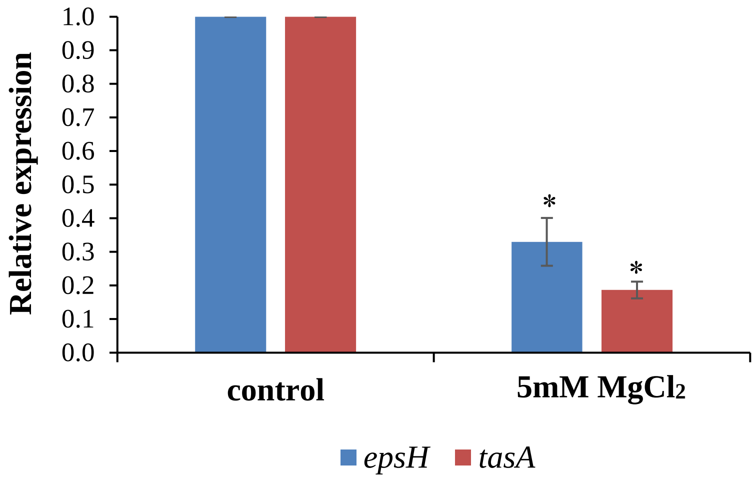

Figure S3 | Effect of Mg^{2+} ions on matrix-genes expression in *B. subtilis*. The real-time RT-PCR analysis was performed for quantitation of *epsH* and *tasA* genes expression in *B. subtilis* cells as described in Methods. *p-value < 0.05 for comparison with control. Error bars represent standard deviation (s.d.).

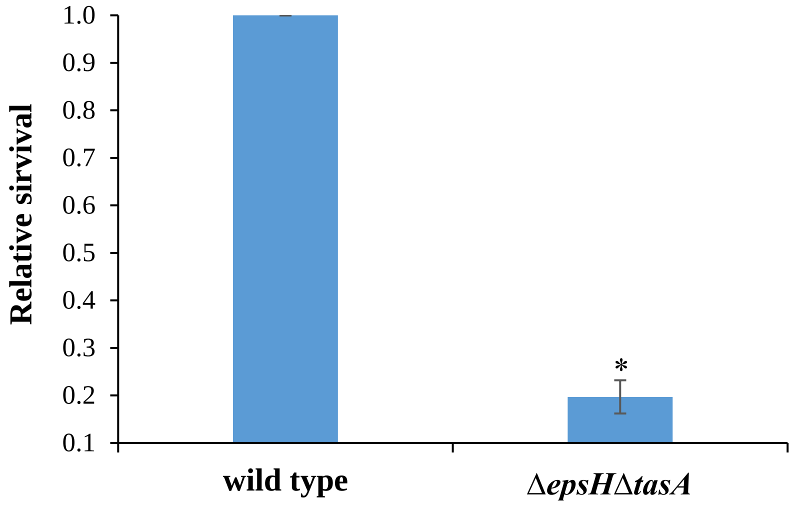

Figure S4 | The effect of heat treatment on *B. subtilis* survival in milk. The *B. subtilis* wild type and Δ*epsH*Δ*tasA* double mutant (which is unable to form biofilm) strains were grown in milk and subjected to heat treatment (at 63°C for 3 min). The relative survival rate of the mutant strain compared to wild type was calculated based on the CFU method. *p-value < 0.05 for comparison with the wild type. Error bars represent standard deviation (s.d.).

Table S1 | Strains used in this study

	Strain	Genotype	Reference
B. subtilis	NCIB3610	undomesticated WT strain	[1]
B. subtilis	YC161	P_{spank}-*gfp* in 3610, CM^R	[2]
B. subtilis	YC189	P_{tapA}-*cfp* in 3610, $Spec^R$	[3]
B. subtilis	DI104	Δ*abrB*, P_{spank}-*gfp* in 3610, Kan^R	Shemesh M. lab
B. subtilis	DI100	Δ*sinR*, P_{spank}-*gfp* in 3610, Kan^R	Shemesh M. lab

B. subtilis	DI103	Δ*epsH*Δ*tasA* in 3610	Shemesh M. lab
B. cereus	ATCC10987	WT strain	Gift from Gohar M. lab
B. licheniformis	MS310	isolated from a dairy farm in Bet Zeid	Shemesh M. lab

Table S2 | Primers used in this study

Strain	Primer	Sequence (5' to 3')
B. subtilis	*sigA*-F	AGATTGCTCAAGAGCCGGTA
	sigA-R	GCGTGGTCAGAAGGTGAAGT
	tasA-F	CCGCTCCTGAATATGATGGT
	tasA-R	GCCGTTCCACTGTGTAGCTT
	epsH-F	ACTCTGACATTGCCCAAACC
	epsH-R	GCCCTGAAGCTGAAAAACTG

Supplementary references

1. Branda, S.S., González-Pastor, J.E., Ben-Yehuda, S., Losick, R. & Kolter, R. Fruiting body formation by *Bacillus subtilis*. *Proc. Natl. Acad. Sci. USA* **98,** 11621–11626 (2001).
2. Chai, Y., Norman, T., Kolter, R. & Losick, R.M. Evidence that metabolism and chromosome copy number control mutually exclusive cell fates in *Bacillus*

subtilis. *EMBO J.* **30,** 1402–1413 (2011).

3. Chai, Y., Chu, F., Kolter, R. & Losick, R. Bistability and biofilm formation in *Bacillus subtilis*. *Mol. Microbiol.* **67,** 254–263 (2008).
